# Application Prospect of Protein-Glutaminase in the Development of Plant-Based Protein Foods

**DOI:** 10.3390/foods11030440

**Published:** 2022-02-02

**Authors:** Xiao Liu, Chao Wang, Xinwen Zhang, Guoqiang Zhang, Jingwen Zhou, Jian Chen

**Affiliations:** 1 Science Center for Future Foods, Jiangnan University, Wuxi 214122, China; liuxiao@jiangnan.edu.cn (X.L.); wangchao@jiangnan.edu.cn (C.W.); gqzhang@jiangnan.edu.cn (G.Z.); zhoujw1982@jiangnan.edu.cn (J.Z.); 2 State Key Laboratory of Food Science and Technology, Jiangnan University, Wuxi 214122, China; zhangxinwen@jiangnan.edu.cn

**Keywords:** protein-glutaminase, deamidation, functional properties, plant-based protein foods, alternatives

## Abstract

Plant-based protein foods as suitable alternative protein sources have recently received increased global interest. The scientific community is exploring effective modification approaches to enhance the functionality of plant-based proteins for expanded utilization. Deamidation has shown great potential for structural modifications and improving the processing efficiency of proteins. In this review, we firstly revisit the enzyme reaction mechanism of protein-glutaminase and its fundamental differences from other enzymatic methods for the deamidation of proteins. Then, the latest advances regarding the suitability of protein-glutaminase modifications for improving the functional properties (e.g., solubility, emulsifying and foaming properties, flavor, and reduction in allergenicity) of plant-based proteins are overviewed. Finally, we address the potential prospect associated with the use of protein-glutaminase in plant-based protein foods, such as meat, dairy, and egg alternatives. This review provides a novel perspective for the design of plant-based protein foods by using protein-glutaminase in order to match animal counterparts in taste and texture, and to fuel widespread adoption.

## 1. Introduction

Protein is essential as it provides the body with amino acids for building cells and repairing tissues. Adequate and high-quality protein intake is particularly important for our health [1]. Compared with plant-based proteins, animal-based proteins in the diet, such as meat, dairy, and egg, account for more water and land resource use and are the second highest source of global greenhouse gas emissions [2]. With an increased global focus on being climate friendly, plant-based protein foods have a fundamental role in achieving and sustaining net-zero emissions in the agriculture sector [3]. Although it is not necessary or recommended to completely avoid animal protein ingredients in food, shifting towards plant protein-based dietary patterns can be beneficial for the health of both consumers and the planet [4].

However, it is worth noting that most plant-based proteins with high contents of glutamine residues induce the aggregation and precipitation of proteins by hydrophobic or hydrogen bond interactions [5], which limit their use and application in the food industry [6]. Protein deamidation mainly occurs through the transformation of the amide side chains of asparagine and glutamine into negatively charged carboxyl groups via the release of ammonia, so as to stretch the structure of the protein and improve the solubility of the protein [7]. In terms of using deamidation to improve the functional properties of plant-based proteins, enzymatic modification of proteins can be carried out under mild conditions, which has great advantages over other treatments [8]. For example, chemical approaches to deamidation, such as hydrothermal treatment under acidic [9] or alkali conditions [10], and/or with anions [11], can cause unfavorable peptide bond hydrolysis and possible amino acid isomerization/racemization, consequently leading to protein damage or safety issues [7]. Therefore, enzymatic approaches to protein deamidation are widely recognized as the desirable method owing to their high reaction specificity under mild environmental conditions and food safety [12].

Protein-glutaminase (EC 3.5.1.44) is a new type of protein-deamidating enzyme that catalyzes the deamidation of glutamine residues in substrate proteins or polypeptides into glutamic acid, which also releases ammonia [13]. Additionally, protein-glutaminase acts on a single substrate and would therefore not be expected to act on other constituents in the food [14]. The negative charge of the glutamyl residue contributes to the electrostatic repulsion of proteins and enables food manufacturers to increase the solubility of proteins, improving their flavor-enhancing properties and techno-functional attributes within a food matrix [6,15]. Protein-glutaminase treatment can also avoid excessive hydrolysis caused by chemical and other protease treatments [7]. Enzymatic deamidation by protein-glutaminase can enhance the physical and chemical properties of plant-based proteins, which may open opportunities for researchers to design plant-based protein foods which are comparable to animal counterparts in appearance, texture, and flavor.

The objective of this review is to update the comprehensive and concise overview of the field of protein-glutaminase application in plant-based protein foods. Research progress in the field of protein-glutaminase and its potential applications in plant proteins is highlighted. Finally, emerging research directions for the prospect of protein-glutaminase application in plant-based protein foods are discussed. This review is expected to forecast that plant-based foods (e.g., meat, dairy, and egg alternatives) could match animal proteins in taste and texture by using protein-glutaminase.

## 2. Basic Knowledge of Protein-Glutaminase and Plant-Based Protein Foods

**Figure 1:**
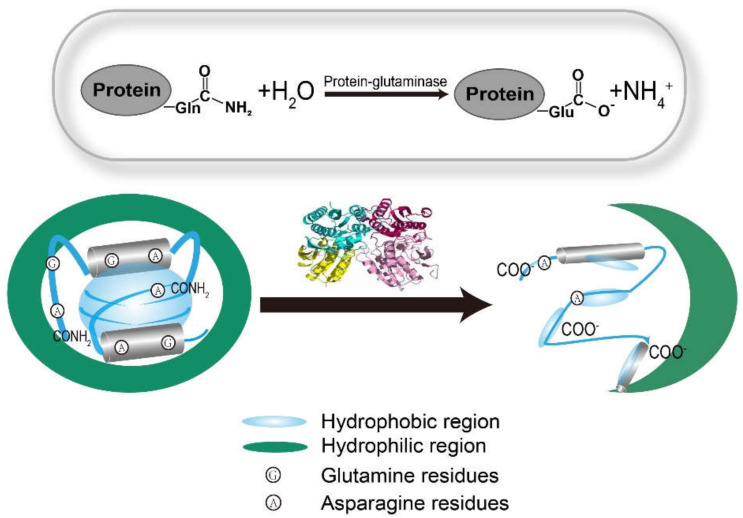
Protein deamidation mechanism by protein-glutaminase.

Protein-glutaminase (EC 3.5.1.44) as a deamidation enzyme was first obtained from the soil bacterium *Chryseobacterium proteolyticum* strain 9670T [16]. Deamidation by protein-glutaminase can lead to the exposure of hydrophobic sites that were previously hidden and convert the amide group to a carboxyl group, which significantly reduces intra/intermolecular hydrogen bonding and enhances electrostatic repulsion between protein molecules (Figure 1). Additionally, protein-glutaminase only reacts with glutamine residues in the side chains of proteins or short peptides and does not react with free glutamine or asparagine residues [17]. Furthermore, there are some disadvantages of other enzymatic deamidations such as peptide-glutaminase (EC 3.5.1.43) and glutaminase (EC 3.5.1.2). On the one hand, the substrate of peptide-glutaminase is limited to short peptides [18], which is not applicable to peptides and proteins. On the other hand, glutaminase specifically catalyzes the deamidation of glutamine residues in proteins or protein hydrolysates, during which slight protein hydrolysis may also occur simultaneously [19]. Therefore, protein-glutaminase is an ideal enzyme for catalyzing the deamidation of proteins. The Food and Drug Administration (FDA) responded to a Generally Recognized as Safe (GRAS Notice NGRN 267) notification submitted by Amano with no questions regarding protein-glutaminase preparation from *Chryseobacterium proteolyticum*.

**Figure 2:**
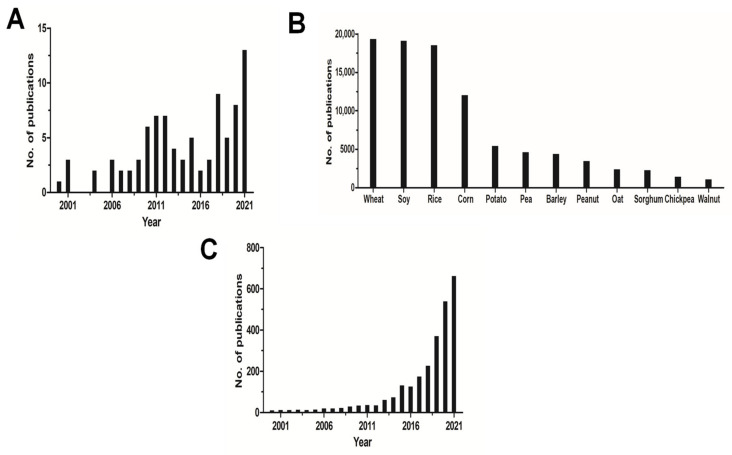
Number of papers published related to (**A**) protein-glutaminase (from 2000 to 2021), (**B**) major sources of plant-based proteins (from 2016 to 2021), and (**C**) “plant-based meat or dairy or egg” (from 2000 to 2021), from a search of the Web of Science database.

We collected data of research publications from the databases of the Web of Science (Figure 2). The topic of protein-glutaminase was first proposed in 2000, and there have been many publications about protein-glutaminase production and its related applications, especially in the last ten years (Figure 2A). In terms of the research topic, researchers mainly focused on heterologous expression systems for protein-glutaminase production. However, there is not much research on its application in agricultural food science, especially surrounding the improvement of protein properties. Unlike other enzymatic deamidations, the use of protein-glutaminase to modify plant proteins is still in its infancy, but the positive results have already gained increasing interest in academic research. In the field of plant protein research, plant-based protein foods (e.g., meat, dairy, and egg alternatives) are more environmentally friendly in their production process and use less of the earth’s resources than traditional livestock [20]. Consumers’ appreciation of plant-based foods stems from a desire for healthy living and a sense of responsibility for environmental issues and animal welfare. Plant-based foods are no longer seen as being only for those living a vegan lifestyle. The popularity of plant-based products continues to rise, which has become a new consumer trend in the food industry. In terms of the number of publications about plant-based proteins in the last five years, the top three were about wheat, soy, and rice (Figure 2B). A search on the Web of Science using “plant-based meat or dairy or egg” as the topic, since the 21st century, led to 2636 results, of which 2434 (>92%) were published in the past ten years (Figure 2C). It is obvious that research on both protein-glutaminase and plant protein-based foods has developed rapidly in the last two decades. The use of protein-glutaminase for improving the physicochemical and functional properties of plant proteins is expected to expand their utilization in plant-based protein foods.

## 3. Deamidation by Protein-Glutaminase to Improve Functional Properties of Plant-Based Proteins

### 3.1. Challenges of the Utilization of Plant-Based Proteins in Food Products

Industrial extraction techniques for plant-based proteins include alkaline extraction, precipitation at the isoelectric point, and harsh spray drying, which lead to protein denaturation and aggregation, resulting in a decrease in emulsifying and foaming stability [21]. Plant-based proteins also contain anti-nutritional compounds with a strong off-taste and tend to have inferior functionality compared to animal-based proteins since they are more difficult to process and more susceptible to extrinsic factors including temperature, pH, and ionic strength [22]. Additionally, some plant-based proteins with a variety of allergens may have a limited scope of utilization [23]. Therefore, it is essential to find an appropriate method to improve the functional properties of plant-based proteins, so as to meet the growing demand for hybrid, clean-labeled, and healthier food products.

### 3.2. Solubility

**Figure 3:**
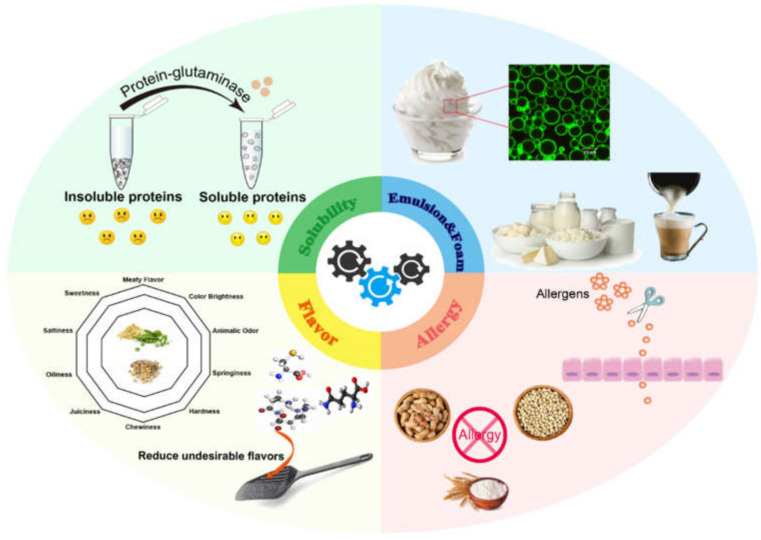
Deamidation by protein-glutaminase to improve the functional properties of plant-based proteins.

Solubility is a prerequisite for plant-based proteins to play a role as a functional component in food and is also the key determinant of their emulsifying and foaming properties, and application in the food industry [24]. Protein-glutaminase can convert glutamine residue in proteins and peptide chains into glutamic acid residue, resulting in an increase in the number of negatively charged carboxyl groups. Previous studies report that the solubility of plant-based proteins (e.g., soy or coconut protein) induced by protein-glutaminase displayed time-dependent changes [25,26]. With a prolonged reaction time in protein-glutaminase treatments, the degree of deamidation (DD) of proteins increased. The increase in negative charges could increase the electrostatic repulsion between molecules, which would reduce intra- and intermolecular hydrogen bonding interactions, finally weakening the aggregation ability of molecules and increasing the solubility of proteins in water (Figure 3). For plant-based protein deamidation by protein-glutaminase, the solubility of non-deamidated and deamidated proteins also shows different sensitivities to environmental stress conditions such as pH and temperature. Compared with non-deamidated samples, the solubility of wheat gluten (WG) in neutral (pH 7) systems was remarkably improved even after a very short deamidation time (1 h, DD 22%), but the behavior of the solubility showed a downward trend under acidic conditions (pH 3) [27]. Yong et al. [28] reported the performance of protein-glutaminase treatment in the solubility of the highly insoluble α-zein. The solubility of α-zein at both pH 5 and pH 7 after deamidation significantly increased to above 80%, even though there was little change under pH 3 conditions. The result of solubility with respect to changes in pH is due to the decline in the isoelectric point of the resulting deamidated protein; thus, the solubility increases in neutral systems and decreases in acidic conditions. Additionally, in the case of oat proteins, both the solubility of the native protein and the low DD (15% and 42%) of the protein at 50 °C were much higher than those at 21 °C. However, for oat proteins with a high DD (59%), a high temperature of 50 °C did not increase their solubility. Therefore, protein-glutaminase treatment may not require additional increases in temperature to improve the solubility of plant-based proteins [29].

### 3.3. Emulsifying and Foaming Properties

Deamidation can modify the structure and hydrophobicity of proteins, which shows great potential for controlling the hydrophilic–hydrophobic balance, while the improvement in amphiphilicity contributes to the formation of a stable layer at the oil–water interface, improving the emulsifying capacity. Compared with the control α-zein at pH 7, the emulsification performance of α-zein treated with protein-glutaminase was effectively improved. At pH 5, deamidation greatly enhanced the solubility of the protein, but the stability of the emulsion decreased [28]. In fact, solubility is not a prerequisite for improving emulsion stability, as the electrostatic repulsion on the oil droplet surface and steric hindrance between the adsorbed protein layers are more important. Kunarayakul et al. [26] found that, in comparison to deamidated coconut proteins at 6 h with a lower DD, deamidated coconut proteins at 12 h with a higher DD showed a higher emulsifying activity index (EAI) and a lower emulsifying stability index (ESI). However, the decline in the value of the ESI could be due to the fact that a high DD could lead to excess net charge and increase the electrostatic repulsion, thus leading to a decline in the interaction between protein and protein. A similar phenomenon can be seen with the foaming properties of soy protein isolates (SPIs) by enzymatic deamidation using protein-glutaminase. The foaming capacity of SPIs was improved after protein-glutaminase modification, but the foaming stability decreased. Generally, appropriate deamidation can enhance the solubility and surface hydrophobicity of plant-based proteins, while improvements in the amphiphilic structure help proteins adsorb at the interface, so as to improve the stability of the emulsion and foam and their potential as a good stabilizer (Figure 3). Therefore, it is necessary to find the optimum conditions of plant-based protein deamidation using protein-glutaminase in order to maintain the best balance between emulsifying/foaming capability and stability.

### 3.4. Flavor

Plant-based proteins, especially legume and oilseed proteins, can easily have an undesired off-flavor which then affects the sensory quality of the food [30]. These undesirable flavor components are mainly aldehydes and ketones produced by the oxidation of unsaturated fatty acids. These carbonyl-containing compounds interact with soy proteins, thereby leading to flavor fade, which influences the flavor of protein foods [31]. The interaction between the flavor compound and the protein can be reversible (noncovalent) or irreversible (covalent). Some noncovalent linkages include hydrophobic and electrostatic interactions, hydrogen bonds, and van der Waals forces [32], while the major covalent linkages include the Schiff base of dithioacetals formed by amino acids or sulfhydryl groups reacting with aldehydes [33]. Flavor–plant protein interactions can be regulated by protein modification. The deamidation of soy protein by protein-glutaminase reduces the overall flavor-binding affinity of the soy protein to the carbonyl-containing vanillin and maltol as common flavor compounds used in the industry [34,35], resulting in a reduction in undesirable flavors (Figure 3). The change caused by protein-glutaminase in the flavor-binding properties of proteins is due to binding from hydrophobic interactions, covalent bonding (Schiff base formations) to weaker van der Waals forces, or hydrogen bonding. Additionally, for soymilk as a soy protein-containing beverage system, protein-glutaminase deamidation has the potential to produce a flavored soymilk with a decreased flavor fade problem [36].

### 3.5. Reduction in Allergenicity

Many plant-based proteins, especially cereal grain substances in the diet, have an allergenic potential which enormously reduces the food choice for allergy sufferers [37]. The Gln-Gln-Gln-Pro-Pro sequence is considered to be the primary binding site of immunoglobulin E (IgE) in wheat protein [38]. Modification of a single type of amino acid (glutamine) is quite effective in reducing the allergenicity of orally administered wheat proteins. An ELISA experiment demonstrated that during the enzymatic deamidation of WG by protein-glutaminase, the DD of WG was increased, and its allergenicity gradually decreased [30]. Therefore, it can be proved that the deamidation effect of protein-glutaminase can be used as a good means to reduce the allergenicity of plant-based proteins (Figure 3).

## 4. The Application Prospect of Protein-Glutaminase in Plant-Based Meat, Dairy, and Eggs

**Figure 4:**
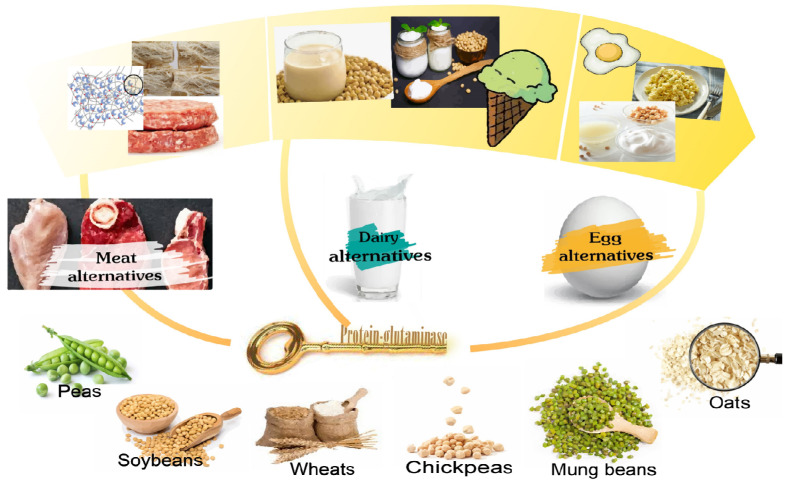
Application prospect of protein-glutaminase in plant-based meat, dairy, and eggs.

By 2050, the global food system will need to meet the dietary needs of more than 10 billion people. With the declining areas of arable land and the depletion of fresh water, it is unsustainable to use animal-based foods as the main source of protein [39]. Consumers’ appetites for plant-based protein foods as alternative proteins are growing as animal-free proteins emerge as a healthy choice. In recent years, plant-based protein foods have morphed from a niche product to a mainstream phenomenon. Improving the taste and texture of plant-based proteins to reach parity with animal-based proteins can help to increase consumer acceptance. Enzymatic modification by protein-glutaminase has been proved to improve solubility, emulsifying/foaming capability, and other functional properties of proteins (Table 1). Protein-glutaminase treatment can contribute to the true texture and authentic flavors of meat, dairy, and egg alternatives (Figure 4).

### 4.1. Plant-Based Meats

Soy, pea, and wheat protein ingredients are the main materials used to create plant-based meats [43]. However, legume-based (e.g., soy and pea) protein foods are still not accepted by consumers owing to the presence of an undesirable bitter and quite beany taste, which provides consumers with an unpleasant experience. Furthermore, vanillin and maltol as model carbonyl-containing flavor compounds can interact with SPIs and lead to flavor fade. Flavor fade problems can be regulated by controlling flavor–protein interactions. The partial deamidation of SPIs by protein-glutaminase can improve their functional properties and decrease the overall flavor-binding affinity of proteins [34], which may reduce undesirable flavors when added to texturized plant-based meats. It is worth noting that protein deamidation is an effective structural modification method for glutamine-rich plant-based proteins, and even a small amount of deamidation can greatly improve the functional properties of proteins; however, it may cause the protein to hydrolyze and break the disulfide bonds of cystine. Compared to other enzymatic approaches to deamidation, protein-glutaminase only deamidates glutamine residues in intact proteins or peptides and does not cause excessive hydrolysis of the protein. Large disulfide-linked protein structures are maintained well after protein-glutaminase treatment. WG as a cystine-rich protein can form an elastic three-dimensional network through covalent cross-linking of disulfide bonds. Thus, the addition of WG has been shown to be more beneficial to the formation of meat alternatives with a fibrous structure and a higher degree of chewiness [44]. One previous study reported the effects of the soy protein concentrate/WG mass ratio (WG 0~30 wt%) on the formation and structure of extruded meat alternatives [45]. The results showed that the highest degree of disulfide cross-linking was observed when the mass ratio of WG increased to 30 wt%, which contributed to the fibrous microstructures in plant-based meats. Furthermore, the popularity of meat alternatives may lead to an increase in the consumption of wheat or soy proteins, which may cause allergic reactions even in people who have never had symptoms of allergic reactions before. As mentioned above, deamidation by protein-glutaminase is an effective strategy to reduce the allergenicity of wheat proteins. Taken together, the reasonable use of protein-glutaminase is expected to improve and solve the texture, flavor, and allergenicity of plant-based meats.

### 4.2. Plant-Based Dairy

Compared with dairy, the protein content of many commercial non-dairy milks is relatively lower or even close to void. With oat drinks, as a popular plant-based dairy alternative, in the process of enzymatic hydrolysis of raw oat materials, in addition to amylase, protease can also be used to improve the content of water-soluble oat proteins. However, the hydrolysis of proteins using protease leads to the formation of low molecular peptides, which have a negative effect on the sensory characteristics of beverages. Protein-glutaminase does not involve substantial protease (peptidase) activity, and with an increase in the DD during protein-glutaminase modification, the soluble proteins in oat drinks are increased. The improvement in oat protein solubility was also reported by a previous study [29].

At present, lattes are one of the most popular coffee drinks and are composed of espresso, steamed milk, and milk foam. The micrometer scale of milk foam produces a smooth and velvety oral perception. Compared with dairy milk, some non-dairy milks can make a decent foam, but once they are introduced to the hot coffee, the microstructure and stability of the foam formed by plant protein-based milks are relatively weakened. To counter this, most barista-specific nut or grain milks include additional natural suspensions and stability regulators. Protein-glutaminase has been shown to induce partial unfolding of plant-based proteins, which contributes to improving the foaming capability and stability of many plant-based proteins. Among raw plant protein materials, the pretreatment of non-dairy milk using protein-glutaminase is conducive to effectively regulating and improving the foamy structure, which opens up a world of possibilities for your next frothy non-dairy latte.

### 4.3. Plant-Based Eggs

Aquafaba, the drained liquid separated from a can or a pot of boiled chickpea seed, can be whipped like egg whites into a meringue or swapped out for eggs entirely in baked goods [46]. The unique functional properties depend on the composition of aquafaba protein, soluble or insoluble carbohydrates, and polysaccharide–protein complexes. The protein content of aquafaba has been found to positively correlate (r^2^ = 0.95) with the foaming capacity of the aquafaba [47]. The partial hydrolysis of proteins by protein-glutaminase can also cause an increase in the solubility and foaming capacity of plant proteins in an aqueous solution. Protein-glutaminase treatment has positive influences on the formation of aquafaba foam (chickpea cooking water). Additionally, mung bean protein isolates are the main ingredient in commercially available plant-based eggs due to their custardy, gelatinous properties. The thickening, emulsification, and water holding capacity of mung bean proteins render egg alternatives as effective humectants and extend the shelf life of bakery products. Therefore, the preparation of plant-based eggs requires improvement in the emulsification and water holding capacity. A deamidation reaction can expose the functional groups within the protein, such as amide and carboxyl groups, and the increment in the protein’s net charge facilitates interactions with water, which results in an increase in its water holding capacity after deamidation. Protein-glutaminase treatment is one solution that provides a higher water holding capacity and emulsification ability, which can be used as an egg substitute to improve the quality of baked goods.

## 5. Conclusions and Outlook

To date, plant-based proteins have been increasingly used as alternatives to animal proteins because of their beneficial effects on health and the environment. The use of protein-glutaminase can contribute to improving the functional properties of plant proteins. The enzymatic deamidation method using protein-glutaminase has potential benefits for producing plant proteins with better processing performance and consumer acceptance, as well as being more important and advantageous compared to other enzymatic approaches to deamidation. In terms of plant-based protein foods, protein-glutaminase treatment has become a positive tool for solubility enhancement and the interfacial stabilization of emulsions and foams, contributes to the production of hypoallergenic plant-based protein foods, and solves the problem of flavor fade in aqueous protein foods.

Although this study finds that protein-glutaminase-modified plant proteins have great market prospects, the current research is still at the laboratory scale due to the low enzymatic performance and yield. Therefore, for there to be large-scale production within the food industry, it is necessary to reduce costs by developing an efficient expression system for high-yield protein-glutaminase production. Additionally, the mechanism and reaction position of protein-glutaminase deamidation in a weak acidic system or in a real plant-based meat, dairy, or egg system still need to be studied, and a protein-glutaminase kinetic model of the action process is required to make it more applicable within the food industry. Overall, the application prospect of protein-glutaminase in the development of plant-based protein foods will emerge in the coming years.

## Figures and Tables

**Table 1:** Improvement of plant-based proteins by means of protein-glutaminase and its potential applications in plant-based meat, dairy, and eggs.

Functional Property	Plant-Based Proteins	Potential Applications in Plant-Based Meat, Dairy, and Eggs
Solubility	Soy protein isolate [25]	More soluble proteins in plant-based dairy products
	Coconut protein [26]	
	Wheat gluten [27,40]	
	α-zein [28]	
	Oat protein [29]	
	Evening primrose seed cake protein [41]	
Emulsifying property	Coconut protein [26]	Plant-based eggs with a higher stability
	Wheat gluten [27]	
	α-zein [28]	
	Oat protein [29]	
	Soy protein isolate [25,42]	
Foaming property	Soy protein isolate [25]	Improvements in the foamy structure of plant-based proteins in non-dairy lattes
	Coconut protein [26]	
Flavor	Soy protein isolate [34]	A reduction in the undesirable flavor of plant-based meat and dairy
	Coconut protein [35]	
	Soymilk [36]	
Reduction in allergenicity	Wheat gluten [27]	Preparation of protein-based meats with low allergenicity

## Data Availability

Not applicable.
